# Conceptual validation of a family-centered intervention for adults with type 1 and type 2 diabetes mellitus: insights from focus groups with healthcare professionals, patients, and families

**DOI:** 10.1186/s12875-025-03133-0

**Published:** 2026-01-06

**Authors:** Vânia Lídia Soares, Carlos Alberto da Sequeira, Maria Carminda Morais, Maria do Céu Barbieri-Figueiredo

**Affiliations:** 1 Abel Salazar Medical Sciences Institute, University of Porto, Porto, Portugal; 2 RISE-Health, Porto, Portugal; 3 School of Nursing of Porto, University of Porto, Porto, Portugal; 4 Health Study and Research Center of the University of Coimbra, CEISUC- CIBB, Coimbra, Portugal; 5 School of Health, Polytechnic of Viana do Castelo, Viana do Castelo, Portugal; 6 Health Sciences Research Unit Nursing, UCISA: E, Coimbra, Portugal; 7 University of Huelva, Campus de “El Carme”. Avda. Tres de Marzo, s/n, Huelva, 21071 Huelva Spain

**Keywords:** Focus group, Conceptual validation study, Program development, Family, Diabetes mellitus, Adults, Qualitative research

## Abstract

**Background:**

Diabetes mellitus is a global health challenge requiring sustained self-management. Evidence highlights the role of families in supporting adults with diabetes mellitus, yet many interventions fail to adopt structured, theory-informed, and family-centred approaches. To address this gap, the psychoeducational intervention “*Juntos com a Família + Capazes”* (Together with Family + Capable) was developed within the Medical Research Council Framework for Complex Interventions, integrating empirical evidence, theoretical models, and stakeholder input. This study conducted a pre-intervention conceptual validation, exploring perspectives of healthcare professionals, adults with Type 1 and Type 2 diabetes mellitus, and family members on its structure, content, and feasibility.

**Methods:**

A qualitative descriptive design was applied. Focus groups interviews with healthcare professionals, adults with diabetes mellitus, and family members, were guided by a semi-structured interview guide. Sessions were audio-recorded, transcribed, and analysed using deductive qualitative content analysis with independent manual coding and consensus discussions.

**Results:**

Three focus groups were held (*n* = 19): two with healthcare professionals and one with adults with diabetes and family members, separated due to participant availability and feasibility constraints. Participants critically reflected on the pre-implementation conceptual design of the programme, described as a six-month intervention with a six-month follow-up, structured into seven modules and delivered flexibly across 7–12 sessions. They endorsed the overall structure and content while suggesting refinements to module sequencing and module titles. Participatory strategies, tailored motivational phone calls, and strong family involvement were considered essential for engagement, sustained behavioural change, and feasibility. Analysis revealed both convergent themes and stakeholder-specific perspectives, integrating clinical expertise with lived experience.

**Conclusions:**

The *“Juntos com a Família + Capazes”* programme was conceptually validated, prior to implementation, as a family-centred, theory-informed, and adaptable intervention for adults with diabetes mellitus and their families. This pre-implementation phase enables refinements to content, sequencing, language, and delivery strategies, strengthening cultural relevance, acceptability, and feasibility. Conducting conceptual validation before piloting ensured that both clinical expertise and lived experiences shaped the intervention, highlighting the value of focus groups in identifying adjustments that enhance its real-world applicability. Future research should evaluate its effectiveness, sustainability, and scalability across healthcare contexts.

**Supplementary Information:**

The online version contains supplementary material available at 10.1186/s12875-025-03133-0.

## Background

Diabetes mellitus (DM) is a global health emergency, affecting approximately 537 million adults worldwide and imposing a major economic and societal burden [1, 2].

Effective diabetes mellitus management requires complex and sustained self-care activities that often occur within the family environment [3, 4]. These demands affect not only the person with DM but also their relatives, with family dynamics strongly influencing adaptation to disease challenges, adherence to self-care, and overall well-being [5–8].

Althought international guidelines increasingly recommend family-centered care, few interventions operationalise this approach in structured, theory-driven ways [9]. Existing programmes often overlook families as active partners in care, despite evidence that their involvement can improve glycaemic control, adherence, emotional well-being, and family resilience [1, 10–13]. From this perspective, family involvement should be understood both as a means to improve self-management and as an end in itself, resulting in more cohesive, informed, and resilient family units [8, 13].

Psychoeducational interventions, by combining medical, behavioural, and psychosocial dimensions, offer a promising strategy to strengthen family participation in chronic disease management [10, 14, 15]. However, few interventions have been conceptually validated to ensure cultural relevance and feasibility before real-world implementation [9].

**Fig. 1:**
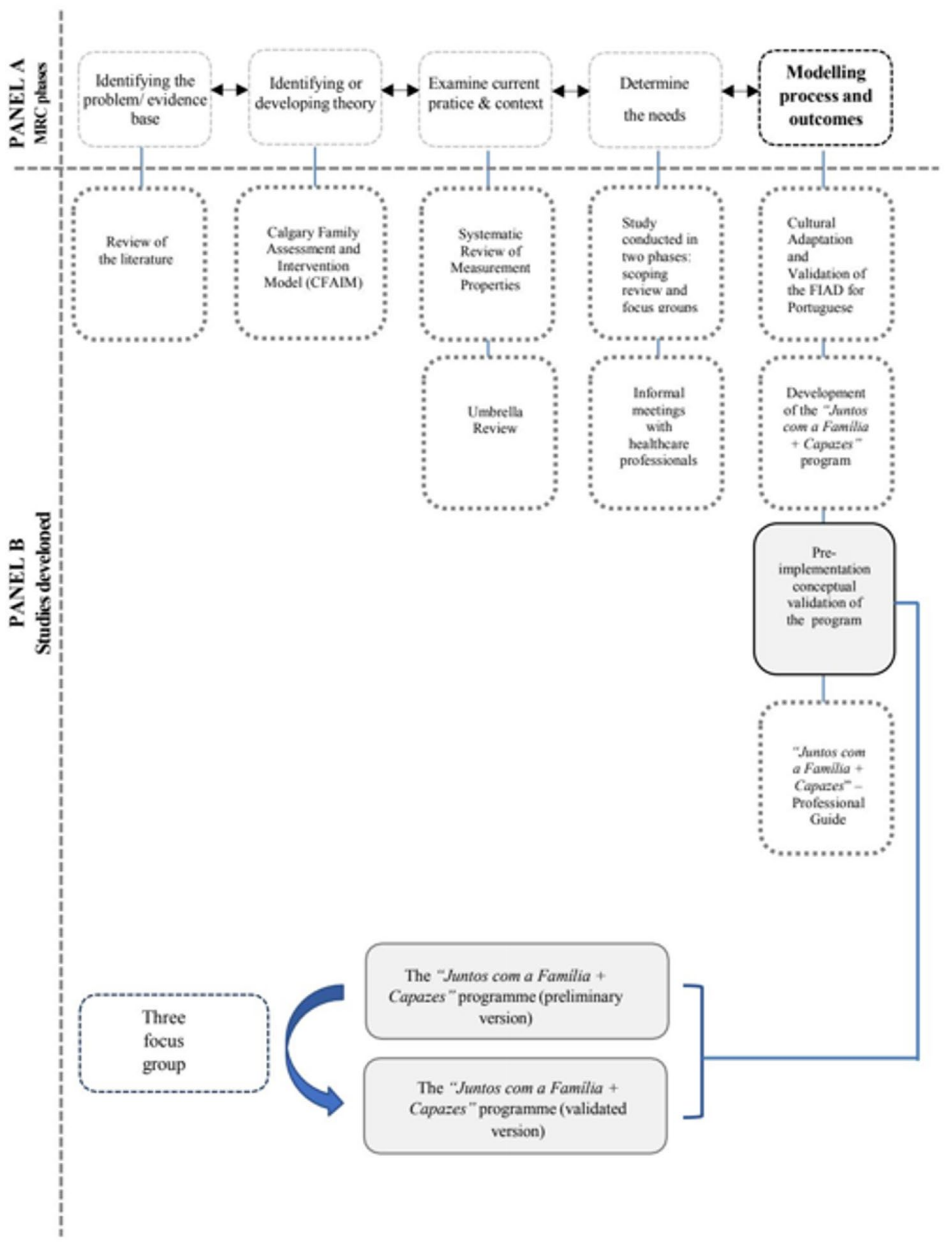
Position within the 2021 MRC Framework and Design of the Pre-intervention Conceptual Validation Study. Abbreviations: MRC: Medical Research Council Framework for Complex Interventions. **A** Positions of this study within the MRC Framework specifically within the Modelling process and outcomes stage of the Development phase.** B** Schematic representation of the pre-intervention conceptual validation study of the “Juntos com a Familia + Capazes” programme

In response, the structured, theory-driven family-centred psychoeducational intervention “Juntos com a Família + Capazes” (Together with Family + Capable) was developed as part of the first author’s doctoral research. Its development followed the Medical Research Council Framework for Complex Interventions, was grounded in the Calgary Family Assessment and Intervention Models (CFAIM), and was informed by empirical evidence, needs assessments, and systematic reviews of the literature, involving adults with type 1 and type 2 diabetes mellitus [8, 9, 16–20]. Figure 1, Panel A, illustrates the position of this study within the MRC Framework, clarifying its role in the intervention development process [19, 20].

The intervention is designed as a six months psychoeducational programme with six-month follow-up, comprising seven mandatory modules, implemented through 7–12 thematic group sessions tailored to participants’ needs, through participatory strategies, reinforced by motivational phone calls and, when feasible, digital tool [21–25]. A detailed description of the intervention is available in a separate manuscript currently under review in a Q1 journal [18].

Our study aims to conduct a pre-implementation conceptual validation of the *Juntos com a Família + Capazes* programme, exploring the perspectives of healthcare professionals, adults with Type 1 and Type 2 DM, and their family members regarding its structure, content, and feasibility. Participants were invited to critically reflect on its acceptability, relevance, and applicability in real-world settings, thereby ensuring that the intervention is both theoretically robust and aligned with lived experiences prior to piloting.

## Method

### Study design and ethics

This qualitative study employed focus group interviews to conduct a pre-implementation conceptual validation of the family-centred psychoeducational intervention “*Juntos com a Família + Capazes”* (Together with Family + Capable). Conducted during the modelling phase of the intervention development MRC Framework for Complex Interventions, the study aimed to refine the programme’s structure, content, and feasibility based on stakeholder input prior to implementation [8, 16, 17]. Figure 1, Panel B, provides an overview of the study design for this pre-implementation conceptual validation.

Focus groups were chosen because they enable interaction and debate, facilitating the emergence of shared and divergent perspectives that are particularly valuable when validating the acceptability and coherence of complex interventions before implementation [26, 27]. It also reflects a deliberate shift from traditional paternalistic care models towards a collaborative, family-partnered approach to chronic disease management [2].

Ethical approval for this study was granted by the Ethics Committee of Alto Minho Local Health (Decree-Law No. 97/95 of 10 May, revised by Decree-Law No. 80/2018 of 15 October). Participants received comprehensive information about the study’s purpose, methods, and potential benefits. Confidentiality and anonymity were strictly maintained, and participation was entirely voluntary, with the option to withdraw at any time without consequences. Written informed consent was obtained from all participants before the focus group sessions.

## Participants and recruitment

To ensure a comprehensive and inclusive understanding of the intervention, purposive sampling was used to recruit three key stakeholder groups: (i) adults with Type 1 and Type 2 diabetes mellitus; (ii) family members actively involved in diabetes mellitus management, and (iii) multidisciplinary professionals with experience in diabetes mellitus care or family-centred approaches. Including these distinct groups was essential to assess the intervention’s feasibility from both professional and lived-experience perspectives. Each focus group was limited to a maximum of ten participants to ensure a balance between richness of discussion and participants’ comfort in sharing experiences [26].

A mixed-group format was initially planned to foster dialogue across perspectives. However, this was not feasible due to limited digital literacy among some participants and scheduling constraints among professionals. Consequently, two focus groups with healthcare professionals were held online, while one with adults with diabetes mellitus and their families was conducted in person. Although this adaptation influenced group dynamics, it ensured accessibility and created a supportive environment that promoted open discussion within more homogeneous groups.

Adults with diabetes mellitus and their family members were recruited via Family Health Units, where healthcare professionals first contacted potential participants by phone. Eligibility criteria included being ≥ 18 years old, speaking Portuguese, and not presenting cognitive impairment. Family members were identified according to Wright and Leahey’s definition of family [8].

Professionals were recruited through institutional networks and included nurses and physicians with expertise in diabetes mellitus and chronic illness management, as well as researchers experienced in the development, implementation, and evaluation of intervention programmes, particularly regarding their effectiveness on health outcomes and costs.

## Data collection

Three focus groups interviews were conducted between January and May 2024: two with healthcare professionals (online) and one with adults with diabetes mellitus and family members (in person, at a Family Health Unit). Each session lasted 90–120 min, allowing sufficient time for in-depth discussion.

A semi-structured interview guide was developed by the research team, drawing on the Calgary Family Assessment and Intervention Model, relevant literature, and expert input from family nursing and diabetes mellitus care [8]. The guide was pilot tested with one healthcare professional experienced in diabetes mellitus care and one adult living with diabetes to ensure its clarity, relevance, coherence. It explored participants’ perspectives on the structure, content, delivery strategies, and feasibility of the *Juntos com a Família + Capazes* programme, (see Additional file 1). To facilitate meaningful engagement, participants received in advance with a concise written summary of the programme’s objectives, structure, and core components. This preparatory material ensured a shared understanding of the intervention concept and supported active, informed contributions during the focus group sessions.

Focus groups were moderated by the first author, a PhD candidate with clinical and research experience in adult diabetes mellitus care. An independent observer, also a PhD candidate with expertise in qualitative research and chronic illness management, recorded contextual observations and group dynamics using structured field notes. All sessions were audio-recorded with participant consent [26, 28]. At the start of each session, a synthesis of key points from previous discussions was presented to participants for clarification and iterative member checking [29].

### Data analysis

Data were analysed using a deductive qualitative content analysis, guided by Mayring’s methodology, and structured around predefined domains corresponding to the programme’s core components (structure, content, delivery strategies, and expected outcomes) [30]. This approach provided a transparent, rule-governed framework to systematically examine the data while capturing both convergent and divergent perspectives.

All transcripts were manually coded by two independent researchers, with discrepancies resolved through discussion. A third researcher reviewing the resulting categories and subcategories to ensure analytical consistency and appropriate levels of abstraction [30]. Interpretation proceeded iteratively, with meaning units systematically grouped into subcategories and broader categories based on the predefined matrix, while allowing space for emergent insights, particularly those reflecting the lived experiences of adults with diabetes mellitus and their families. Field notes collected during the sessions were incorporated into the analysis to enrich the contextual understanding and capture relevant non-verbal interactions. Illustrative quotations are presented in the Results section and labelled according to participant group: HP (healthcare professional), AWD (adult with diabetes mellitus), or FM (family member), each followed by a numerical identifier (e.g., HP3, FM5), to preserve anonymity while distinguishing perspectives across stakeholder groups.

These systematic procedures reinforced the trustworthiness, credibility, and transparency of the findings, in line with the Consolidated Criteria for Reporting Qualitative Research (COREQ) [31].

## Results

Three focus group interviews were conducted between January and May 2024: two with healthcare professionals (online) and one with adults with diabetes mellitus and their family members (in person, at a Family Health Unit). This separation was due to feasibility constraints (digital literacy and scheduling). Across the three sessions, 310 min of discussion were recorded and fully transcribed, producing 54 pages of text. From this dataset, 591 quotations were initially coded, of which 365 were retained for analysis.

All invited experts (*n* = 19) agreed to take part in the study, and none withdrew. A total of nine multidisciplinary professionals from healthcare and academic settings participated across two focus groups: six in the first and seven in the second, with some attending both sessions. The third focus group included ten participants, comprising five adults diagnosed with diabetes mellitus and five family members.

Professionals had a mean age of 48.7 years and extensive experience in diabetes mellitus or chronic disease management. Adults with diabetes mellitus were predominantly male (mean age 71.6 years), while family members were mostly female (mean age 67.4 years). The detailed sociodemographic characteristics are presented in Table 1.

**Table 1:** Characteristics of focus group participants

Focus Group	*N*	Mean Age (years)	SD	Sex (M/F)	Education Level	Mean Time (years)	SD	Professional/Sociodemographic Characteristics
Professionals	9	48,7	14,9	2 M 7 F	Master (*n* = 2), PhD (*n* = 7)	Experience with chronic Disease: 2,3 Experience with DM: 2,4	0,9 0,8	Nurse (*n* = 3), Physicians (*n* = 1), Full Professor (*n* = 1), Principal Adjunct Professor (*n* = 1), Coordinating Professor (*n* = 2); Investigator (*n* = 1)
Adults with diabetes mellitus:	Type 1 (*n* = 1) Type2 (*n* = 5)	71,6	9,6	3 M 2 F	Primary (*n* = 3), Secondary (*n* = 2)	Time Since Diagnosis: 7,8	6,7	Employed (*n* = 2), Retired (*n* = 3)
Family members	5	67,4	10,3	1 M 4 F	Primary (*n* = 3), Secondary (*n* = 2)	Years of support: 7,8	6,7	Employed (*n* = 2), Retired (*n* = 3)

*Abbreviations:* *DM* Diabetes Mellitus, *SD* Standard Deviation, *F *Feminine, *M* Masculine

Participants reflected on four key dimensions of the “*Juntos com a Família + Capazes”* programme, structure, content, delivery strategies, and expected outcomes, providing both convergent and stakeholder-specific perspectives on its relevance, feasibility, and acceptability. A deliberate strength of this study was the triangulation of perspectives: healthcare professionals contributed expertise on programme structure, feasibility, and anticipated health outcomes, while adults with diabetes mellitus and their family members grounded the validation process in lived experience, ensuring cultural and contextual relevance. To illustrate findings, quotations are included and identified by participant group (HP = healthcare professional; AWD = adult with diabetes mellitus; FM = family member), followed by a numerical code.

**Fig. 2:**
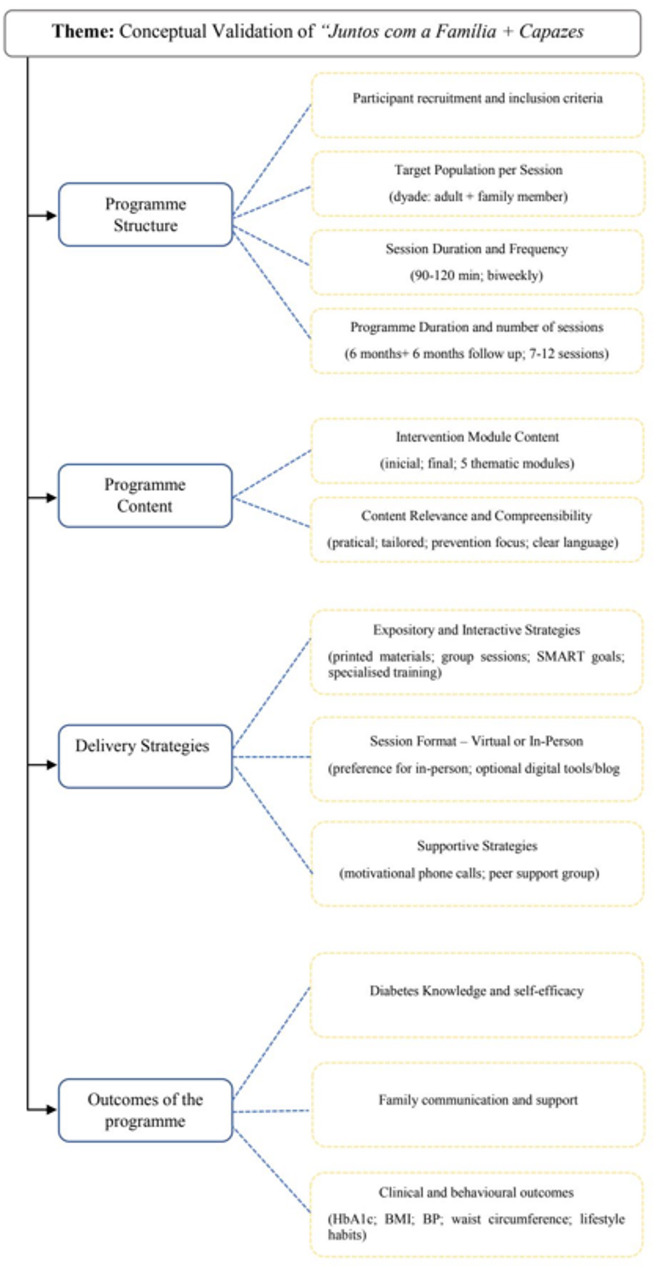
Tree diagram of the categories and subcategories of the intervention. Abbreviations: SMART Specific, Measurable, Achievable, Relevant, and Time-Bound, HbA1c: glycated hemoglobin, BMI: Body Mass Index, BP Blood Pressure

The analysis yelded four main categories and twelve subcategories, which are represented in Fig. 2.

###  Category 1. Programme structure

#### Subcategory 1.1: participant recruitment and inclusion criteria

Participants consistently agreed that recruitment should be undertaken by multidisciplinary healthcare professionals across different levels of care or through self-referral, provided that individuals met the established inclusion criteria. As one professional explained: *“Expanding referral pathways beyond primary care allows for the involvement of a wider range of professionals*,* who can contribute diverse perspectives and expertise to the individuals’ therapeutic education plans.”* (HP4).

These inclusion criteria—being ≥ 18 years old, regularly attending healthcare appointments, speaking and understanding Portuguese, and having no cognitive impairment, were unanimously endorsed for both adults with diabetes mellitus and family members. Several participants stressed that advanced age alone should not be considered an exclusion factor, as long as cognitive capacity was preserved. As one participant stated:

*“(…) Just because someone is older doesn’t mean they can’t participate. I may be old*,* but I still have a clear mind and can think perfectly well.”* (AWD1).

Flexibility was also highlighted as essential to ensure broad dissemination and adaptation to diverse groups. Professionals emphasised that recruitment pathways should not be overly restrictive, allowing coordinators to adapt inclusion according to each group’s needs: *“If we aim for the programme to be disseminated nationwide*,* it cannot be overly restrictive”* (HP10).

All participants also emphasised that flexibility in group composition was essential, suggesting that sessions could be organised by knowledge level or treatment regimen to enhance relevance and peer learning, while still allowing diversity in age and time since diagnosis. This diversity was perceived as valuable for creating a supportive environment, as one participant explained: “…*It is important for them (adults with diabetes and their family members) to feel more comfortable speaking with peers of different ages and disease durations*,* even about issues that are sometimes difficult*,* such as sexuality…”* (HP6).

The inclusion of family members was unanimously supported, provided they were identified by the person with diabetes mellitus as trusted and relevant in managing their health, with recognition that “family” extends beyond biological ties. At the same time, participants agreed that pregnant women with diabetes mellitus should be excluded, given the distinct clinical and psychosocial needs of gestational diabetes mellitus: *“Pregnant women with diabetes are a different case—they have to nourish the baby. I think it would be mixing distinct situations*,* which require specific approaches”* (FM1).

#### Subcategory 1.2: target population per session

All participants (*n* = 19) agreed that each session should involve the dyad, composed of the adult with diabetes mellitus and their family member. This format was perceived as essential for fostering a supportive family environment and avoiding situations where one individual might feel excluded from important information. Professionals emphasised the role of shared participation in building trust within the family unit: *“…it doesn’t make sense to separate the family and the people diagnosed… it could cause some mistrust between them…”* (HP6).

Adults with diabetes mellitus valued learning alongside their relatives, describing how this approach could facilitate understanding and mutual support. As one participant explained: *“…I like things to be explained to me at the same time as they’re explained to my wife*,* because she doesn’t ask questions*,* but I do and so I help her…”* (AWD6).

Family members also reinforced these benefits, highlighting those joint sessions made it easier to share knowledge and collaborate in daily self-management: “…*I think it’s great to have my husband with me (…) it makes it easier for us to understand things*,* and when questions come up*,* we can help each other…*” FM5.

#### Subcategory 1.3: session duration and frequency

Participants expressed diverse opinions regarding session length, and no consensus was reached. About half considered 120 minutes excessive, favouring 90 minutes as a standard, while others argued that 90 minutes was insufficient to cover content and allow meaningful interaction. As one professional noted: *“ … my only concern is the length of each session (…) 120 minutes would be too long…”* (HP8).

In contrast, others stressed the need for flexibility, suggesting that session duration should vary between 90 and 120 min depending on objectives: *“(…) maybe there could be a variation between the sessions*,* that is*,* depending on the session we were doing*,* we could vary between 90 and 120 minutes (…) we could adjust (the duration of the sessions) according to the objectives of each session (…) because there are sessions that have many objectives (…)”* (HP7).

Adults with diabetes mellitus and their family members emphasised that the most important aspect was having enough time to discuss all their questions and doubts: *“…the length of the sessions will depend on our doubts… sometimes there are subjects that are difficult to understand straight away”* (AWD4). This flexibility was ultimately endorsed, with participants agreeing that facilitators should adapt session duration to the specific needs of each group: *“…the programme will be more helpful if it’s adapted to other people’s specific situations and needs…”* (HP6).

Regarding frequency, there was unanimous agreement that sessions should be held every two weeks to minimise travel and accommodate daily responsibilities: *“…Yes*,* twice a month seems reasonable*,* as in-person sessions require travel …and it is not always easy to align sessions with participants’ daily schedules…”* (HP5).

Both adults and family members highlighted the importance of scheduling the entire calendar during the baseline session to facilitate planning: *“… we have to know our life (…) we have to organize ourselves to be able to attend all the sessions (…)”* (FM1).

#### Subcategory 1.4: programme duration and number of sessions

All participants agreed that the proposed six-month intervention period, followed by a six-month follow-up, was appropriate. They felt this this duration was sufficient to consolidate learning and sustain behavioural change: *“(…)yes*,* it seems to me that six months is an adequate time (…)”* (HP5).

Family members likewise supported the proposed timeframe, noting its suitability even in the absence of prior experience with similar programmes: *“ (…) I’ve never taken part in anything like this before*,* but it sounds good to me.”* (FM5).

The integration of motivational phone calls during both phases was also validated. Participants agreed that brief monthly calls would reinforce continuity and provide timely support. As one adult observed: *“ (…) the phone calls are great for clearing up doubts (…) don’t you think it’s at home when the questions usually come up?”* (AWD3). Family members echoed this, emphasising the practicality of short, structured calls: *“(…) Like when we’re shopping and all (…)”* (FM1).

Regarding the number of sessions, all participants endorsed the seven-module structure while supporting flexibility to adjust according to group needs. Professionals recommended a range of seven to twelve sessions, allowing facilitators to adjust the intensity and structure in collaboration with participants: “(…) *so I don’t know if I should leave this number open and give it almost as a recommendation. The program has to have at least seven sessions and can go up to 12 sessions (…)”* (HP7).

Adults and family members highlighted that, regardless of the number, sufficient time must be provided to clarify doubts without disrupting daily responsibilities: *“I don’t really care about how many sessions or modules there are*,* as long as we have time to ask questions and get clear answers.”* (AWD5).

###  Category 2. Programme content

#### Subcategory 2.1: intervention module content

All participants agreed that the proposed module topics were appropriate and consistent with the objectives of the programme. Professionals, drawing on their expertise in diabetes mellitus care, chronic illness management, and family health, confirmed that the modules reflected the main challenges they encountered in practice: *“…It looks good to me; it’s aligned with what we see in our daily work with patients”* (HP8).

Family members also found the content well-structured and relevant: *“(…) I think it’s well put together”* (FM1). Adults with diabetes mellitus reinforced this view, recognising the modules as covering key aspects of disease management: *“(…) it seems to me that it covers the most important things for managing diabetes”* (AWD1).

#### Subcategory 2.2: content relevance and compreensibility

Drawing on their clinical and academic experience, the professionals provided a critical appraisal of the proposed content. They emphasized it should be practical, relevant, and directly applicable to daily life rather than overly theoretical. Professionals agreed that the programme should focus on empowering participants to manage their diabetes mellitus effectively and prevent complications, while being responsive to individual needs and the socio-economic contexts in which they live: *“(…) the program to make a difference and what the literature shows is that the more tailored the programs are to people’s specific needs*,* the more successful they are*,* of course (…)”* (HP5).

Suggestions included refining module titles and sequencing to better reflect lived experiences, as for example, renaming the complications module as “Possible Complications”, in order to emphasise the importance of prevention rather than addressing complications only after they have manifested: *“(…) the logic that this program should have been (…) to prevent complications*,* or rather*,* possible complications*,* because possible gives us the chance of not having them (…)”* (HP4).

Adults with diabetes mellitus emphasised the importance of tailored, concrete guidance, especially regarding diet: *“I don’t even know what to eat anymore (…) everything seems bad for you (…)”* (AWD2). Family members echoed this concern, underscoring the need for clear and accessible language: *“(…) we want more information that helps us*,* but it needs to be easy to understand (…) not complicated language (…)”* (FM5).

Based on the feedback gathered during the focus groups, the intervention programme was revised to include seven mandatory modules: an initial “Module Zero: Baseline”, which serves as an introductory session, and a “Final Module: Closing Session”, designed to consolidate learning and reflect on the experience. The five thematic modules in between address key areas of diabetes mellitus management and family involvement. While all modules are compulsory, their sequence may be adapted collaboratively between facilitators and participants to address group needs and priorities. The final structure and content are detailed in Table 2.

**Table 2:** “*Juntos com a Família + Capazes” *programme: Overview of Module Content, Stakeholder Perspectives, and Adaptations

Module	Content, Objectives& Strategies	Participants' Perspectives	Adjustments Made
Module 0 – Baseline (Initial)	Present the programme and module structure; Define SMART goals group; Describe and assess clinical/non-clinical variables; Emphasise dyad participation and health partnership Support groups via digital platforms (e.g., WhatsApp, Instagram) to foster peer interaction and share information.	Assess family’s specific needs and digital capacities to build trust and ensure accessibility (E,P)	Added digital literacy screening and individual goal-setting; Included a specific needs assessment to tailor the intervention to participants' realities.
Transforming Habits	Promote understanding of healthy lifestyles and their physical, emotional, and social benefits; Explore the role of family and social support, communication, self-management, mental well-being, and community resources in diabetes mellitus care.	Emphasised need for practical, realistic strategies (E,P) Identify family-specific contextual factors that may act as barriers to effective diabetes mellitus management (E)	Included concrete tools for habit tracking and goal planning. Engage participants through collaborative activities and practical games to promote active involvement, while identifying specific learning needs
Balanced Nutrition	Key concepts of balanced nutrition and its role in preventing complications; family meal planning and healthy cooking; food label reading and informed choices; social and gender roles in nutrition; cultural and economic factors influencing food habits; healthy eating outside the home; glycaemic index, weight and waist monitoring; and strategies to recognise and manage hypo/hyperglycaemia.	Emphasised need for practical, realistic strategies (E, P)	Engage participants through collaborative activities and practical games to promote motivation and involvement. Identify specific learning needs and incorporate local food examples to enhance relevance and comprehension.
Movement and Health	Raise awareness of the benefits of regular physical activity and educate on types of exercise, safety precautions, and integration with diabetes mellitus self-management. Define personalised goals, address social and contextual barriers, and consider gender roles and health status in the adoption of active routines.	Highlighted barriers like time and fatigue (E,P); family support seen as essential (E)	Included tips for integrating activity into routines and involving family. Identify local opportunities and integrate community-based physical activity initiatives to enhance accessibility, motivation, and sustained engagement in regular exercise routines.
Family and Communication	Explore family roles, supportive and obstructive behaviours, and communication patterns. Address emotional impact, social determinants, family values, and gender roles. Promote effective communication and conflict resolution to support diabetes management.	Strongly valued (E,P); improved understanding among family members (E).	No major changes; confirmed importance of this module.
Diabetes Mellitus and Complications	Enhance understanding of diabetes mellitus, self-monitoring practices, and strategies for preventing complications.	Title too clinical; it is recommended to minimise the use of biomedical language (P)	Renamed to focus on "Possible Associated Complications" and practical management.
Module Final Session Closing	Aassess clinical/non-clinical variables; Summarise learnings, reinforce goals, plan follow-up.	Valued as opportunities for closure and reinforcement (P); follow-up and motivational phone calls were highlighted as particularly important for maintaining engagement and clarifying doubts (E,P).	No major changes; confirmed importance of this module.

*Abbreviations:* *E* end-users (adult diagnosed with diabetes mellitus/family members,* P* professionals

###  Category 3. Intervention delivery strategies

#### Subcategory 3.1: expository and interactive strategies

All participants (*n* = 19) endorsed the proposed delivery strategies, which combined structured teaching methods, such as verbal presentations and the distribution of printed educational materials, with interactive approaches, designed to actively engage participants in the learning process, including group discussions and problem-solving exercises. Professionals emphasised that combining both formats was essential to ensure knowledge acquisition and practical application. They also valued the collaborative setting of (Measurable, Achievable, Relevant, and Time-Bound (SMART) goals, while recommending that participants be given the option to define individual goals with facilitators in one-on-one moments outside the group.

The pivotal role of facilitators was underlined, with participants stressing the need for professionals trained in both diabetes mellitus self-management and group facilitation, and for regular coordination meetings to reinforce the family-centred approach: *“ (…) absolutely (…)important to invest in training professionals. They need to be trained in diabetes self-management and group facilitation (…)”* (HP6).

Adults with diabetes mellitus and family members highlighted the value of interactive and engaging sessions, contrasting them with more passive teaching: *“(…) I really enjoy talking and asking questions (…) diabetes brings a lot of doubts*,* and the games will be fun”* (AWD1); *“(…) just listening all the time isn’t great (…) it feels like school and it makes you sleepy”* (FM1).

Together, these perspectives reinforced the importance of a balanced approach, combining structured content delivery with participatory learning to foster motivation, meaningful dialogue, and practical skills for self-management.

#### Subcategory 3.2: session Format – Virtual or In-Person

The possibility of incorporating digital platforms, social media pages, or WhatsApp groups generated extensive discussion. Professionals, drawing on prior experiences, cautioned that such approaches often face barriers related to participants’ resources and digital literacy: *“(…) from my experience*,* it doesn’t work with most groups (…)”* (HP5). Similar concerns were voiced by adults and family members, who reported difficulties using computers or modern mobile phones and expressed discomfort with online communication: *“(…) I don’t like that (…) those computers and talking from a distance”* (AWD3); *“(…) my wife and I struggle with computers and more modern mobile phones (…)”* (FM1).

Despite these barriers, participants highlighted the importance of timely reminders—preferably by text or phone call, to encourage consistent attendance: *“It’s important that they send text messages to remind us of the training days and times (…) so everything is in order. Or call us”* (FM1).

In light of these findings, in-person sessions were prioritised as the preferred format. Digital tools for content reinforcement were considered only if participants demonstrated sufficient access and literacy, introduced in Session Zero (Baseline). Additionally, some participants suggested a dedicated blog as a complementary resource to compile information across modules.

#### Subcategory 3.3: supportive strategies

Participants strongly endorsed the inclusion of motivational phone calls during both the intervention and follow-up periods, recognising their value for maintaining engagement, clarifying doubts, and sustaining motivation: *“ (…) the phone calls are really important for clearing up doubts and having someone to talk to about my condition (…)”* (AWD5).

Professionals highlighted the importance of keeping these calls brief, structured, and guided by motivational interviewing principles: *“ (…) these calls shouldn’t be too long…15 minutes seems appropriate during the intervention*,* and no more than 20 minutes during the follow-up period. This kind of reinforcement really makes sense (…)”* (HP7).

Professionals and families also emphasised the need for continuity mechanisms, such as follow-up contacts and access to supportive resources, to consolidate programme gains. In addition, the creation of online peer support groups was suggested as a complementary strategy, although concerns regarding digital literacy and equitable access led participants to recommend their implementation only when groups show readiness and interest. If implemented, such platforms should be moderated and aligned with the programme’s goals, ensuring that the information shared remains accurate and constructive.

Taken together, the delivery strategies were considered appropriate and feasible, combining structured learning with personalised support and flexible formats. These mechanisms were viewed as essential to sustaining participant motivation and ensuring that the programme could effectively achieve its intended outcomes, both at individual and family levels.

#### Category 4. Outcomes of the programme

Outcomes of the programme were grounded in the programme’s theoretical framework and refined through participant input, ensuring alignment with its objectives and expected impact. Across all focus groups, there was unanimous agreement on the anticipated benefits.

#### Subcategory 4.1: diabetes mellitus knowledge and self-eficaccy

Across all groups, the programme was expected to enhance understanding of diabetes mellitus, including including aspects such as diet, medication, complications, and practical self-care strategies. Adults and family members stressed the value of having a space to clarify doubts together: “ (…) *we need a space to ask questions and get answers (…)”* (FM5). They also highlighted the importance of joint learning to support change at home: *“…if we learn together*,* we can also change small things at home…”* (AWD1). For some, knowledge was directly linked to self-efficacy and the prevention of complications: *“(…) I want to feel that I can manage this better (…) and that I won’t go blind or lose a leg like my neighbour (…)”* (AWD4).

Professionals reinforced this perspective, emphasising the critical role of health literacy in enhancing autonomy and self-efficacy among adults with diabetes mellitus and their families in managing the disease: *“ (…) for me*,* this is a fundamental aspect that a programme like this should aim to achieve (…)”* (HP4).

#### Subcategory 4.2: family communication and support

Participants expected the programme to strengthen family communication, thereby improving family functioning, increasing confidence in disease management, and ultimately enhancing quality of life for both adults with diabetes mellitus and their relatives. Involving the family was seen as essential to fostering mutual support and shared responsibility, which could help reduce the emotional burden often associated with the condition.

Professionals stressed the importance of assessing communication, noting its association with stress, depressive symptoms, and treatment adherence: *“ (…) for me*,* these are fundamental aspects to assess because we know that diabetes has a strong positive association with stress and depressive symptoms (…)”* (HP6); *“(…) the way people communicate within the family*,* whether there is support for the person diagnosed or not*,* makes a significant difference in how well they adhere to the therapeutic plan”* (HP5).

Adults with diabetes mellitus and their family members echoed this view, highlighting challenges in dialogue and the impact on daily routines: *“(…) sometimes it is not easy to talk to her about her food choices (…)”* (FM4); *“(…) he is my father*,* so I have to respect him (…) but he does not listen to my advice about diabetes”* (FM5); “(…) *sometimes*,* if he didn’t argue with me and just came for a walk with me*,* it would be better. But he only tells me what to do*,* and I have so many chores at home that I just don’t feel like it (…) I do everything on my own (…)”* (AW1).

#### Subcategory 4.3: clinical and behavioral health outcomes

Participants expected that the programme would lead to measurable clinical improvements typically used to evaluate the effectiveness of interventions in diabetes mellitus care. These included metabolic and cardiovascular indicators such as Body Mass Index (BMI), glycated hemoglobin (HbA1c), waist circumference, systolic and diastolic blood pressure, alcohol consumption (measured in drinks per day), and smoking habits (measured in cigarettes per day). As noted by one: *“(…) those are the health gains expected from a program of this nature”* (HP4). Another participant confirmed: *“(…)* yes, they seem right to me. That’s what the doctor always says (…)” (AW3).

Beyond biomedical markers, participants underscored the importance of behvioural outcomes. These encompassed improved self-management behaviours, such as adherence to treatment, medication compliance, and regular blood glucose monitoring. Participants further anticipated increases in health-promoting behaviours, including physical activity (hours per week) and healthier dietary practices. Such changes were seen as essential to achieving better metabolic control and enhancing overall well-being: *“(…)in my experience with these programs for people with diabetes*,* I believe the main outcomes are indeed these”* (HP9).

Table 2 presents an overview of the final set of programme modules, their objectives, and the adaptations introduced during the conceptual validation process. To highlight the unique contributions of different stakeholders, participants’ perspectives are identified by group (healthcare professionals, adults with diabetes mellitus, and family members). This distinction underscores the value of including end-users alongside service providers in refining the intervention Methodological details are provided in the COREQ Checklist (see Additional file 2).

Together, these findings demonstrate that the conceptual validation process successfully integrated professional expertise with the lived experiences of adults with diabetes mellitus and their families, resulting in an intervention refined for cultural relevance, feasibility, and real-world applicability.

## Discussion

### Main findings

The “*Juntos com a Família + Capazes”* (Together with Family + Capable) programme addresses a critical gap in adult diabetes mellitus care by adopting adopting a structured, family-centred psychoeducational approach. Unlike traditional patient-focused education, this programme positions the family as a unit of care, recognizing their needs, interactions, and roles as integral to effective disease management [2, 9, 13]. This approach aligns with growing evidence that family dynamics, communication patterns, and shared responsibilities influence self-management, treatment adherence, and psychological well-being in chronic illness [6, 32].

Conceptual validation through focus group interviews demonstrated its cultural relevance, feasibility, and acceptability prior to implementation [26, 33, 34]. By engaging healthcare professionals, adults with diabetes mellitus, and family members, the study confirmed that family inclusion is both valued and necessary, reinforcing families not merely as supportive contexts but as units of care. Practical refinements emerged, such as tailoring module sequencing, rephrasing biomedical language to reduce fear, and reinforcing motivational follow-ups [35]. Flexibility, regarding session duration, delivery format, and adaptation to literacy and socio-economic contexts, was highlighted as a core strength of the programme. Notably, participants validated the inclusion of motivational follow-up phone calls and the option of digital reinforcement when feasible, but prioritised in-person sessions given barriers of access and digital literacy, particularly among older adults [36, 37].

#### Comparison with existing literature

The emphasis on family inclusion and psychoeducational approaches to improve outcomes, such as adherence, communication, and psychological well-beinh, is consistent with prior literature [6]. However, few complex interventions in diabetes have undergone rigorous theoretical development or conceptual validation, which may explain inconsistent effectiveness in trials [38]. Many randomised controlled trials move directly to pilot or effectiveness phases without a modelling phase, leaving important gaps in pre-implementation evaluation [39]. By contrast, this study contributes to filling that gap. Grounded in the CFAIM and MRC Framework, it presents a conceptual validation of a family-centred intervention, triangulating the perspectives of healthcare professionals, adults with diabetes, and family members [8, 16]. This approach ensured that refinements were not only theoretically sound but also grounded in lived experience [39, 40].

Although conceptual validation and stakeholder co-construction have been explored in other chronic conditions (e.g., cardiovascular disease, nutrition interventions) they remain rare in diabetes research, where interventions often struggle with adherence and scalability [38, 41]. Our findings suggest that conceptual validation prior to pilot testing enhances cultural relevance, acceptability, and feasibility, addressing these well-recognised implementation barriers [26, 33].

#### Tailoring group composition and training modalities

A key insight from the focus groups was that heterogeneous needs among participants may limit comparability of outcomes if not systematically addressed. To mitigate this, the programme begins with a baseline assessment using the CFAIM, covering structural, developmental, and functional domains [8]. This evaluation guides facilitators in tailoring group composition to pedagogically meaningful criteria, particularly diabetes-related knowledge or therapeutic regimen, rather than age or time since diagnosis alone.

In practice, grouping participants with similar levels of knowledge, even if differing in age or disease duration, may foster peer learning through the exchange of diverse lived examples [42]. Similarly, organising groups by treatment regimen (e.g., oral therapy versus insulin-based regimens) can enable targeted discussions around specific techniques and safety issues, while mixed groups may help demystify injectable therapies and stimulate collaborative problem-solving [42, 43]. Importantly, this flexibility also recognises that younger and older participants, and their families, may require differentiated training modalities to ensure engagement, comprehension, and practical applicability across life stages [42, 43].

To ensure fidelity and replicability, explicit criteria for group composition and thresholds for knowledge levels will be described in future implementations [31]. Anchoring this tailoring process in the CFAIM framework ensures responsiveness to participants’ needs while maintaining transparency and consistency [8]. By reframing heterogeneity not as a barrier but as a resource for co-learning, the programme enhances both adaptability and transferability across diverse care settings.

### Implications for practice

The study underscores the importance of positioning families as active partners in diabetes care [8]. Health professionals should consider family involvement not as optional but as an integral component of therapeutic education [8, 13]. The “*Juntos com a Família + Capazes”* (Together with Family + Capable) programme offers a structured, adaptable framework that could be integrated into primary care and community health services, with potential to improve self-efficacy, family functioning, and adherence. Furthermore, the findings provide practical insights into tailoring delivery methods, prioritising face-to-face interaction while strategically integrating digital tools to extend reach without excluding vulnerable groups [44, 45].

The intervention’s grounding in the CFAIM provided a systematic framework to capture family structure, development, and functioning [8]. An important implication is that each group will be constituted after a baseline family assessment, enabling facilitators to tailor sessions to participants’ needs and specificities [8, 14].

### Strengths and limitations

This study has several strengths. First, it followed COREQ guidelines to ensure methodological rigor and transparency [31]. Purposive sampling maximised diversity in gender, age, professional and experiential background, and socioeconomic status, thereby enhancing transferability. Credibility was reinforced through triangulation of perspectives, combining healthcare professionals’ expertise with the lived experiences of adults with diabetes mellitus and their families. Dependability and confirmability were supported by systematic analytic procedures, including independent coding, consensus resolution, and external review. Reflexivity was maintained through journaling and team dialogue, allowing researchers to critically examine preunderstandings shaped by their backgrounds in diabetes care, family health, and qualitative research [31].

Nonetheless, some limitations must be acknowledged. As a pre-implementation conceptual validation study, findings reflect stakeholders’ anticipated perceptions rather than lived experiences of the intervention in practice. This reliance on hypothetical reflections may restrict the generalisability of conclusions about feasibility and acceptability [31]. The imbalance between participant groups (two focus groups with professionals versus one with adults and family members) could also be viewed as a limitation, though triangulation mitigated this by ensuring complementary insights. Focus groups facilitated dynamic interaction but may have limited exploration of deeply personal experiences, while the mix of online and face-to-face sessions introduced variability in group dynamics, though it improved accessibility [28, 46]. Despite these limitations, the combination of methodological rigor, reflexivity, and stakeholder diversity strengthens the validity and trustworthiness of the findings and ensures that the resulting programme is both conceptually robust and contextually relevant.

## Future research

The conceptual validation confirmed that the “*Juntos com a Família + Capazes*” programme is acceptable, feasible, and responsive to stakeholder needs, providing a solid foundation for pilot testing. Future research should evaluate its effectiveness in improving health outcomes, and family functioning. Longitudinal studies are needed to assess sustainability and cost-effectiveness, while implementation research should explore scalability across healthcare systems with varying resources. Comparative studies could also examine the transferability of this approach to other chronic illnesses. Finally, research should explore strategies for integrating digital tools in ways that extend reach without exacerbating inequalities in access.

## Conclusion

The “*Juntos com a Família + Capazes*” programme was conceptually validated as a family-centred and theory-informed intervention for adults with diabetes mellitus and their families. This pre-implementation phase refined content, structure, and delivery strategies, ensuring cultural relevance, acceptability, and feasibility. By integrating both clinical expertise and lived experiences, the study demonstrated the value of conceptual validation in tailoring interventions to heterogeneous family needs before pilot testing. Future research should now evaluate its effectiveness, sustainability, and scalability across healthcare contexts to inform the integration of family-centred psychoeducational programmes into diabetes care. Importantly, this positions the programme as one of the first conceptually validated family-centred psychoeducational interventions in diabetes specifically developed and validated within the Portuguese healthcare context, addressing a critical gap in pre-implementation evaluation.

## Supplementary Information

Supplementary Material 1.

Supplementary Material 2.

## Data Availability

The datasets used and/or analyzed during the current study are available from the corresponding author on reasonable request.

## Abbreviations

AWD: Adult with diabetes mellitus
CFAIM: Calgary Family Assessment and Intervention Models
COREQ: Consolidated criteria for reporting qualitative research
DM: Diabetes mellitus
FM: Family member
HP: Healthcare professional
MRC: Medical research council
SMART: Specific, Measurable, Achievable, Relevant, and Time-Bound
