# Supplementary material for: Conceptual validation of a family-centered intervention for adults with type 1 and type 2 diabetes mellitus: insights from focus groups with healthcare professionals, patients, and families
Source: BMC Prim Care. 2026 Jan 6;27:71. doi: 10.1186/s12875-025-03133-0 (PMC12930986; doi:10.1186/s12875-025-03133-0)
Supplement: Supplementary file 1 — Supplementary Material 1. [file 12875_2025_3133_MOESM1_ESM.docx]

Additional file 1: Interview Guide For Focus Group Sessions

| **Itema** | **Role of the Moderator** |
| --- | --- |
| Introduction | Thank participants for their willingness to participate.  Invite participants to introduce themselves. |
| **Legitimising the Focus Group** | Request permission to record the session.; Ensure that the recording will remain confidential and used solely for analysis purposes; Guarantee anonymity of individual responses; Emphasise the importance of everyone’s contributions to the discussion. |
| Development | Present the aim and purpose of the *“Juntos família + Capazes”* (Together with Family + Capable) psychoeducational programme  Briefly outline the development phases of the programme.  Present the specific objectives:   - To explore participants' perceptions regarding the structure of the programme; - To explore participants' perceptions of the programme content; - To explore participants' views on the proposed methodology;   Propose a collective evaluation of the session |
| Conclusion | Thank the group for their collaboration;  Reaffirm adherence to ethical principles, particularly regarding data confidentiality;  Inform participants that access to the results will not be available after the completion of the study. |

| **Main Objective 1:** To identify the defining characteristics of a psychoeducational nursing intervention programme for individuals with type 2 diabetes and their families, with regard to its structure | | |  |
| --- | --- | --- | --- |
| **Specific Objectives** | **Key Questions** | **Topics for Exploration** | |
| To explore participants' perceptions of the programme’s structure | What is the appropriate duration of the programme?  What should be the duration of each session?  What is the ideal length for follow-up phone calls?  How long should WhatsApp and Facebook group access be maintained?  How many sessions should be included in the programme?  What is the ideal frequency of sessions?  Who should participate in each session type? | – What duration do you consider adequate for the overall programme?  – What duration should each session have?  – Should session duration vary depending on the delivery format (in-person vs Zoom)?  – What is the ideal duration for phone contacts during the intervention and follow-up?  – For how long should access to WhatsApp and Facebook groups be maintained?  – How many sessions and phone contacts should be included in the programme?  – What should be the interval between sessions and phone contacts?  – Who should participate in in-person and Zoom sessions? | |

| **Main Objective 2:** To identify the defining characteristics of a psychoeducational nursing intervention programme for individuals with type 2 diabetes and their families, with regard to its content. | | |  |
| --- | --- | --- | --- |
| **Specific Objectives** | **Key Questions** | **Topics for Exploration** | |
| To explore participants' perceptions of the programme content | What topics should be addressed in a programme developed for adults with diabetes and their families? | – What contents should be included in a psychoeducational intervention programme?  – Do you agree with the topics proposed across the sessions?  – Is the distribution of topics across sessions appropriate?  – Should additional topics be considered?  – Are the contents suitable for in-person, Zoom, WhatsApp, and Facebook group sessions?  – Should there be separate WhatsApp and Facebook groups for people with diabetes and family members?  – Is peer sharing and communication through digital platforms appropriate and useful? | |

| **Main Objective 3:** To identify the defining characteristics of a psychoeducational nursing intervention programme for individuals with type 2 diabetes and their families, with regard to its methodology. | | |  |
| --- | --- | --- | --- |
| **Specific Objectives** | **Key Questions** | **Topics for Exploration** | |
| To explore participants' perceptions of the implementation strategies | What are the most appropriate strategies to implement the intervention?  Which methodologies foster greater participation from individuals and families?  Are motivational strategies important?  Are follow-up strategies important? | - Are the strategies proposed for Zoom and in-person group sessions appropriate? - Is the use of interactive games appropriate? - Do you find the use of WhatsApp and Facebook groups beneficial? - Do these platforms enhance health outcomes? Should these platforms remain active during the follow-up period? - What strategies promote the engagement of individuals and families? - Are motivational strategies (e.g., phone calls) appropriate? - Are follow-up strategies (e.g., phone calls) appropriate? – Is a 6-month follow-up period adequate? | |
